# RETRACTION: Application of Continuing Nursing Intervention on Wound Infection and Ulcers in Patients With Diabetic Foot: A Meta‐Analysis

**DOI:** 10.1111/iwj.70446

**Published:** 2025-03-26

## Abstract

**RETRACTION:** LiX.‐X. , XuJ. , ChenJ. , GaoF. , WangQ.‐J. , and YangS.‐H. , “Application of Continuing Nursing Intervention on Wound Infection and Ulcers in Patients With Diabetic Foot: A Meta‐Analysis,” International Wound Journal 21, no. 2 (2024): e14692, 10.1111/iwj.14692.PMC1082270442052987

The above article, published online on 28 January 2024, in Wiley Online Library (http://onlinelibrary.wiley.com/), has been retracted by agreement between the journal Editor in Chief, Professor Keith Harding; and John Wiley & Sons Ltd. Following an investigation by the publisher, all parties have concluded that this article was accepted solely on the basis of a compromised peer review process. The editors have therefore decided to retract the article. The authors did not respond to our notice regarding the retraction.

**RETRACTION:** X.‐X. Li , J. Xu , J. Chen , F. Gao , Q.‐J. Wang , and S.‐H. Yang , “Application of Continuing Nursing Intervention on Wound Infection and Ulcers in Patients With Diabetic Foot: A Meta‐Analysis,” International Wound Journal 21, no. 2 (2024): e14692, 10.1111/iwj.14692.PMC1082270442052987

The above article, published online on 28 January 2024, in Wiley Online Library (http://onlinelibrary.wiley.com/), has been retracted by agreement between the journal Editor in Chief, Professor Keith Harding; and John Wiley & Sons Ltd. Following an investigation by the publisher, all parties have concluded that this article was accepted solely on the basis of a compromised peer review process. The editors have therefore decided to retract the article. The authors did not respond to our notice regarding the retraction.

